# COVID-19 팬데믹이 임부의 우울에 미치는 영향

**DOI:** 10.4069/kjwhn.2023.02.21.2

**Published:** 2023-03-31

**Authors:** Da-bin Seok, Hyeon Ok Ju

**Affiliations:** College of Nursing, Dong-A University, Busan, Korea; 동아대학교 간호학부

**Keywords:** COVID-19, Depression, Mental health, Physical distancing, Pregnant women, 코로나바이러스병-19, 우울, 정신건강, 사회적 거리두기, 임부

## Abstract

**Purpose:**

Uncertainty and restrictions on daily life have increased fear, stress, and depression during the coronavirus disease 2019 (COVID-19) pandemic. Depression is the most common mental health problem in pregnant women. The purpose of this study was to evaluate the levels of fear and stress related to COVID-19 experienced by pregnant women, as well as their levels of depression, and to examine the factors associated with depression during pregnancy.

**Methods:**

This was a cross-sectional, correlational study conducted among 153 pregnant women who visited a maternity hospital in Busan, South Korea. A self-reported questionnaire was used for data collection from December 18, 2021 to March 8, 2022. Data were analyzed using descriptive statistics, the independent t-test, one-way analysis of variance, Pearson correlation coefficients, and multiple regression.

**Results:**

Pregnant women experienced a moderate level of fear related to COVID-19, with an average score of 21.55±4.90. The average score for depression during pregnancy was 14.86±11.10, with 50.3% of the participants experiencing depression (≥13). The factors associated with depression during pregnancy were fear of COVID-19, contact with a confirmed case of COVID-19, being in the third trimester of pregnancy, high stress levels due to difficulties experienced from social distancing measures, and unintended pregnancy. These five statistically significant factors explained 35.0% of variance in depression during pregnancy.

**Conclusion:**

Considering the prevalence of depression in pregnant women during the COVID-19 pandemic, it is necessary to develop interventions to reduce anxiety by providing correct information and alleviating the stress of social distancing.

## Introduction

임신은 여성의 삶에서 신체적, 정서적, 사회적 변화를 경험하는 민감한 사건으로[1], 여성과 태아는 다양한 심리적 요인에 의해 영향을 받는다[2]. 그중 우울은 임부에게서 가장 많이 나타나는 정신건강 문제이다[3]. 임부는 임신으로 인한 호르몬 변화로 입덧과 체중 증가 등 신체 증상을 겪으며 임신 유지와 태아 건강에 대한 걱정으로 불안을 경험하고 심한 경우 우울을 경험할 수도 있다[4]. 우울은 임부의 활동, 영양, 수면 양상을 변화시키고, 임부의 기분과 태아의 발달에 영향을 미치며, 유산, 조산, 저체중아 출산 및 출생 시 낮은 Apgar 점수 위험을 증가시킨다[5,6].

2019년 12월 중국 후베이성 우한시에서 원인 불명의 폐렴이 발생했다는 보고를 시작으로 코로나바이러스 감염증 2019 (coronavirus disease 2019, COVID-19)가 확산하였고[7], World Health Organization (WHO)는 2020년 3월 11일 세계적 대유행을 의미하는 팬데믹(pandemic)을 선포하였다[8]. WHO와 전 세계 보건당국은 COVID-19의 확산을 통제하기 위한 일환으로 국가적 수준에서 ‘사회적 거리두기’와 격리를 권고하며[9], 감염 확산 정도에 따라 다중이용시설의 영업시간 및 수용 인원을 제한하였고, 개인적 수준에서는 마스크 착용, 손 위생과 주변 환경 소독, 백신 접종 등의 예방 행동을 권고하였다[10]. 2021년 12월 중증도는 다소 낮으나 감염력과 전파력이 높은 오미크론(Omicron) 변이 바이러스가 국내로 유입되었고, 2022년 1월부터는 오미크론 확산세가 전국적으로 지속되었다. 방역 당국은 확진자가 급증함에 따라 의료체계 유지를 위해 입원 중심 치료에서 재택중심 치료로 전환하는 대책을 시행하였고 3차 백신 접종을 권고하였다[11].

COVID-19 팬데믹은 임부의 일상에 영향을 미치는 심리적 스트레스 요인으로 나타났으며[9,12], 사회적 거리두기와 격리 등의 사회적 조치는 바이러스 감염으로부터 보호하기 위한 방역지침이지만 임부의 정신건강에는 부정적인 영향을 미치는 것으로 나타났다[6,12]. 2019년 1월부터 2020년 9월까지 임부의 정신건강에 대해 조사한 연구들의 메타 분석에 따르면 COVID-19 팬데믹 동안 임부의 우울과 불안은 각각 31%와 37%로 높게 나타났다[13].

일반적으로 임부는 면역력이 저하된 상태로 호흡기 바이러스 감염에 의한 폐렴이 발생하면 폐기능이 급격하게 악화되어 중환자실 입실과 기계적 환기 적용 가능성이 높고 중증 질환 발생 비율이 증가한다[14]. 그리고 임부의 호흡기 바이러스 감염은 임신중독증, 태아곤란과 같은 임신 합병증 발병률을 높이며 조산 및 제왕절개 분만을 초래할 수 있다[15]. 그러나 팬데믹 발생 초기에 COVID-19 감염과 사망자가 지속적으로 증가하고 통제할 수 없는 국제적 보건 위기 상황에서 임부는 백신 안정성이 확보되지 않아 접종이 제한적이었고[14], 태반을 통한 수직감염 정보도 부족한 실정이었다[15,16]. 이에 임부는 임신 유지 및 태아에게 미치는 영향에 대한 염려와 바이러스에 대한 두려움을 보였고[17], COVID-19에 대한 두려움은 임부의 우울을 유발하는 것으로 나타났다[18].

COVID-19 스트레스는 다차원적이고, 문화적 환경에 따라 다른 특성이 있으며, 한국인을 대상으로 한 연구에 의하면 3가지 요인으로 나타났다[19]. 주변 환경 접촉을 통해 본인이 COVID-19에 감염되거나, 이로 인해 주변 사람들에게 영향을 미치는 것 등에 대한 두려움, 사회적 거리두기 방역지침으로 일상 생활 및 문화 생활, 사회적 대면 접촉이 줄어들면서 경험하는 어려움, 그리고 방역지침을 준수하지 않고 확산의 원인이 되는 특정 상황 및 개인에 대한 분노 등이며, 특히 타인에 분노의 경우는 서양과 달리 한국의 집단주의적 성향이 반영되어 나타나는 특성이다[19,20].

임부는 COVID-19 팬데믹의 불확실성과 일상의 제한 및 방역지침의 지속적인 변화로 자신이 기대한 주산기 계획과 다른 현실 상황을 마주하고 감염에 대한 스트레스와 출산 준비에 대한 스트레스를 경험한다[21]. 정기적인 산부인과 진료, 산전 교육 참여, 출산을 위한 산후조리 준비가 어려워지는 등의 COVID-19와 관련된 스트레스는 임부의 우울을 증가시키며 정신건강에 부정적 영향을 미치는 주요 요인이었다[22]. 하지만 임부는 심리적 증상을 신체 변화로 인한 피로 증상으로 오인하고 겉으로 표현하지 않는 특성이 있어 산전에 심리적 문제를 선별하는 것을 놓치기 쉽다[23].

국내 임부를 대상으로 COVID-19 유행 상황에서 임부들이 심리적 영향을 어느 정도 받는지, 영향 요인이 무엇인지에 대한 연구는 부족한 실정으로, 신종 감염병 유행 상황에 임부의 심리적 영향과 관련 요인을 파악하여 감염병과 사회적 조치가 임부의 정신건강에 얼마나 영향을 미치는지 규명하는 것은 의의가 있다. 본 연구는 임부를 대상으로 COVID-19에 대한 두려움과 COVID-19 스트레스, 우울 정도를 파악하고, 임부의 우울에 미치는 영향 요인을 확인하여 신종 감염병 유행 시 임부의 정신건강을 위한 간호 중재 개발의 기초자료를 제공하기 위한 것으로, 구체적 목적은 다음과 같다.

(1) 대상자의 우울 수준, COVID-19에 대한 두려움, COVID-19 스트레스 정도를 파악한다.

(2) 대상자의 특성에 따른 우울의 차이를 파악한다.

(3) 대상자의 우울과 COVID-19에 대한 두려움, COVID-19스트레스의 관계를 파악한다.

(4) 대상자의 우울에 미치는 영향 요인을 파악한다.

## Methods

Ethics statement: This study was approved by the Institutional Review Board of Dong-A University (No. IRB-2-1040709-AB-N-01-202111-HR-076-02). Informed consent was obtained from the participants.

### 연구 설계

본 연구는 임부의 우울, COVID-19에 대한 두려움, COVID-19 스트레스 정도를 확인하고, 임부의 우울에 미치는 영향 요인을 파악하기 위한 횡단적 상관성 조사연구이다. 연구의 기술은 STROBE 보고지침(https://www.strobestatement.org/)에 따라 작성하였다.

### 연구 대상

본 연구의 대상자는 COVID-19 유행 상황에 임신을 유지하고 부산에 소재한 2개의 산부인과 전문 병원에서 산전 진찰을 받는 2–3삼분기 임부를 편의 추출하였으며, 설문지를 읽고 응답이 가능하며 본 연구 목적을 이해하고 참여에 자발적으로 동의한 자이다. 임신 1삼분기에는 임신 오조로 인한 신체 증상 및 임신 유지와 태아 기형 유무에 대한 불안감을 가질 수 있어[24], 안정기에 해당하는 2–3삼분기 임부를 대상으로 하였다. 임신 합병증 치료 및 입원으로 인한 우울을 배제하고 COVID-19 팬데믹 상황이 임신 중 우울에 미치는 영향을 확인하기 위해 현재 임신 중 임신 합병증을 진단받고 치료 중이거나 태아의 신체적·구조적 문제를 진단받은 임부, 과거 우울을 포함하여 정신병력이 있는 임부는 제외하였다. 표본의 크기는 G*power program 3.1.9.7 프로그램을 이용하였으며, 분석 방법은 회귀분석으로 유의 수준 .05, 효과 크기 .15 (중간 크기), 검정력 .80, 예측 요인 13개를 입력하여 표본 크기를 산출하였다. 최소 표본 수는 131명이었으나 탈락률 20%를 고려하여 총 164명에게 설문지를 배부하였고, 응답이 미비하거나 불충분한 11명을 제외하고 총 153명(응답률 93.3%)을 대상으로 조사하였다.

### 연구 도구

#### 우울

우울은 Radloff [25]가 개발한 The Center for Epidemiologic Studies Depression Scale (CES-D)를 Eaton 등[26]이 정신장애의 진단 및 통계편람(Diagnostic and Statistical Manual of Mental Disorders, DSM) 제 4판의 우울 삽화 증상 및 기간을 반영하여 개정하고 Lee 등[27]이 번역 및 타당화한 한국판 역학 우울 척도 개정판(Korean Version of Center for Epidemiologic Studies Depression Scale-Revised, K-CESD-R)으로 측정하였다. 이 도구는 한국의 사회‧문화적 특성이 잘 반영되어 우울증 역학 연구에서 유용한 선별 검사이고 공공 자료로 자유롭게 사용이 가능하며[27], 증상의 존재 기간을 기준으로 우울 정도를 측정하는 20개의 문항으로 구성되어 있다. ‘1일 미만(0점)’에서 ‘2주간 거의 매일(4점)’까지의 5점 Likert 척도로, 총 점수 범위는 0점에서 80점으로 점수가 높을수록 우울 정도가 높은 것을 의미하며 최적 절단점 13점 이상은 임상적 우울군에 해당한다[27]. Lee 등[27]의 연구에서 신뢰도 Cronbach’s α는 .98이고, 본 연구에서 Cronbach’s α는 .92였다.

#### COVID-19에 대한 두려움

COVID-19에 대한 두려움은 Ahorsu 등[17]이 개발하고 Seong 등[28]이 한국어로 번안하고 타당도를 확인한 ‘COVID-19에 대한 두려움(Korean version of the fear of COVID-19 scale, FCV-19S-K)’을 사용하였다. FCV-19S-K 도구는 총 7문항으로 ‘전혀 그렇지 않다’ 1점에서 ‘매우 그렇다’ 5점의 5점 Likert 척도로, 합산 점수 범위는 7점에서 35점이고 점수가 높을수록 COVID-19에 대한 두려움이 높음을 의미한다. 개발 당시 도구의 신뢰도 Cronbach’s α는 .82이고, Seong 등[28]의 연구에서 Cronbach’s α는 .87이었다. 본 연구에서 Cronbach’s α는 .76이었고, 본 도구는 개발자와 번안자에게 모두 사용 승인을 받았다.

#### COVID-19 스트레스

COVID-19 스트레스는 COVID-19로 인해 한국인들이 겪고 있는 스트레스를 측정하기 위해 Kim 등[19]이 개발한 ‘COVID-19 Stress Scale for Korean People’로 측정하였다. 이 도구는 ‘감염에 대한 두려움’ 9문항, ‘사회적 거리두기로 인한 어려움’ 6문항, ‘타인에 대한 분노’ 6문항 등 3개의 하위 요인 총 21문항으로 구성되어 있다. 본 도구의 반응 척도는 ‘전혀 그렇지 않다(1점)’에서 ‘매우 그렇다(5점)’까지의 5점 Likert 척도이다. 각 하위 영역의 점수 범위는 평점 최저 1점에서 최고 5점이며 점수가 높을수록 해당 영역에 대한 스트레스를 많이 받음을 의미한다.

Kim 등[19]은 총점을 사용하는 것이 적합할지 3개의 하위 요인을 구분하여 사용하는 것이 더 적합할지 판단하기 위하여 1요인 모형과 3요인 모형을 비교하는 확인적 요인 분석을 시행하였고, 모형의 적합도는 3요인 모형이 1요인 모형보다 우수하였다. 따라서 본 연구에서도 총점이 아니라 하부 요인으로 결과를 분석하였다.

Kim 등[19]의 연구에서 하부 요인의 신뢰도 Cronbach’s α는 감염에 대한 두려움 .93, 사회적 거리두기로 인한 어려움 .81, 타인에 대한 분노 .89였다. 본 연구에서의 하위 영역별 신뢰도 Cronbach’s α는 각각 .84, .76, 85였다. 본 도구는 개발자에게 사용 승인을 받았다.

#### COVID-19 관련 특성

COVID-19 관련 특성은 선행 연구[29,30]를 바탕으로 연구자가 재구성하였으며, COVID-19 확진자 접촉 유무, COVID-19 관련 삶의 제약 유무, COVID-19 관련 정보 수집 매체, COVID-19 관련 정보 신뢰도, COVID-19로 인해 임신 중 겪은 힘들었던 점과 염려되는 점 등 6문항으로 구성되었다. COVID-19로 인해 임신 중 힘들었던 점은 1순위 6점, 6순위 1점으로 배점하였으며, 각 문항의 평균 점수 범위는 1점에서 6점으로 점수가 높을수록 힘들었다는 것을 의미한다. COVID-19 유행으로 임신 중 염려되는 점은 1순위 5점, 5순위 1점으로 배점하였으며, 각 문항의 점수 범위는 1점에서 5점으로 평균 점수가 높을수록 염려 정도가 높았다는 것을 의미한다.

#### 일반적 특성 및 산과적 특성

일반적 특성은 연령, 교육수준, 직업 유무, 월 평균 소득수준 등 4문항, 산과적 특성은 현재 임신기간, 분만력, 계획 임신 여부, 산전 진료, 임신 합병증, 우울 진단 유무, 선호하는 분만 형태 등 총 7문항으로 구성되었다.

### 자료 수집 기간 및 방법

자료 수집 기간은 2021년 12월 18일부터 2022년 3월 8일까지였다. 자료 수집 방법은 부산에 소재하며 응급 분만이 가능한 산부인과 전문병원 8곳 중 연구 진행에 동의해준 2개의 병원 외래에 연구 대상자 모집 공고문을 부착하여 모집하였다. 연구자는 모집 공고문을 통해 자발적으로 참여한 대상자에게 연구의 목적과 방법을 설명하였고, 수집된 자료는 연구의 목적으로만 이용되며 참여 중단을 원할 경우 언제든지 철회할 수 있음을 설명하였다. 이에 동의하고 이해한 대상자에게 서면으로 동의서를 받았고, 밀봉 가능한 봉투에 담아 설문지를 전달하였다. 설문지는 집으로 가져가 작성하도록 하였고 이후 작성 완료된 설문지는 병원 내원 시 밀봉된 상태로 제출하도록 하였으며 해당 병원의 연구 보조원을 통해 연구자가 일괄 회수하였다. 그리고 연구 대상자에게 감사의 표시로 소정의 답례품(5,000원 상당의 커피 쿠폰)을 제공하였다.

### 자료 분석 방법

본 연구의 수집된 자료는 IBM SPSS for Windows ver. 28.0 (IBM Corp., Armonk, NY, USA)을 사용하여 분석하였으며 통계적 유의 수준은 *p*<.05로 설정하였다.

(1) 대상자의 일반적 특성, 산과적 특성 및 COVID-19 관련 특성은 실수, 백분율, 평균과 표준편차로 분석하였다.

(2) 대상자의 우울 정도, COVID-19에 대한 두려움, COVID-19 스트레스 등은 평균과 표준편차로 분석하였다.

(3) 대상자의 특성에 따른 우울은 독립 t-검정(independent t-test)과 일원분산분석(one-way analysis of variance)로 분석하였다.

(4) 대상자의 우울, COVID-19에 대한 두려움, COVID-19 스트레스의 관계는 피어슨 상관계수(Pearson correlation coefficients)으로 분석하였다.

(5) COVID-19 유행 시 임부의 우울에 미치는 영향 요인은 동시적 다중회귀분석(simultaneous multiple regression analysis)으로 분석하였다.

## Results

### 대상자의 일반적 및 산과적 특성과 COVID-19 관련 특성

대상자의 일반적, 산과적 특성은 Table 1과 같다. 대상자의 평균 연령은 32.8세였고, 대부분이 대학교 졸업 이상(92.2%)으로 휴직을 포함하여 직업이 있는 경우가 73.2%였고, 가정의 월 평균 소득 수준은 300만 원 이상 500만 원 미만이 42.5%였다.

대상자의 산과적 특성으로는 임신 2삼분기가 51.0%, 임신 3삼분기는 49.0%였다. 대상자의 과반수 이상이 초임부(70.6%)였고, 계획 임신은 68.0%였다. 대상자의 80.4%는 정기적으로 산전 진료를 받았고, 선호하는 분만 방법으로는 질식 분만이 58.2%였다.

대상자의 COVID-19 관련 특성은 Table 1과 같다. 대상자의 대부분은 COVID-19 확진자와 접촉한 경험이 없으며(84.3%), COVID-19로 인하여 삶의 제약을 경험하였다(92.2%). COVID-19 관련 정보 수집 경로는 미디어(39.9%), 인터넷(35.3%) 순이었으며, 임부의 과반수 이상(71.2%)이 수집한 정보를 신뢰하는 것으로 나타났다.

대상자가 COVID-19 유행으로 불편하고 힘들었던 점은 ‘산전 진료 시 보호자 출입 제한(4.10점)’, ‘외출 제한(3.85점)’, ‘코로나바이러스 백신 접종 제한(3.82점)’ 순이었다. COVID-19로 인하여 가장 염려스러운 사항은 ‘태아에게 영향을 줄까 걱정된다(4.08점)’였고, 다음은 ‘내가 코로나바이러스에 감염될까 걱정된다(3.67점)’, ‘코로나바이러스 감염으로 진료를 받지 못하고 임신 합병증이 생길까 걱정된다(2.58점)’ 순이었다(Table 2).

### 대상자의 우울, COVID-19에 대한 두려움 및 COVID-19 스트레스 정도

본 연구에서 대상자의 우울 정도는 평균 14.86±11.10점이었고, 13점 이상인 임상적 우울 수준의 대상자는 50.3%였다. COVID-19에 대한 두려움 정도는 평균 21.55±4.90점으로 중간 정도 수준이었다. COVID-19 스트레스의 평점 평균을 각 하위 요인 별로 살펴보면, ‘타인에 대한 분노'가 4.16±0.69점, ‘감염에 대한 두려움’이 4.00±0.51점, ‘사회적 거리두기로 인한 어려움’이 3.41±0.69점 순이었다(Table 3).

### 대상자의 특성에 따른 우울의 차이

임부의 특성에 따른 우울의 차이를 살펴보면 임신 3삼분기(t=–2.68, *p*=.008), 비계획 임신인 경우(t=–3.11, *p*=.002), 임부가 COVID-19 확진자와 접촉한 경험이 있는 경우(t=–3.20, *p*=.002), 삶의 제약이 없다고 응답한 경우(t=3.49, *p*=.003)가 우울 점수가 더 높은 것으로 나타났다(Table 1).

### 대상자의 우울과 COVID-19에 대한 두려움, COVID-19 스트레스의 관계

본 연구에서 우울은 COVID-19에 대한 두려움(r=.38, *p*<.001)과 양의 상관관계가 있었고, COVID-19 스트레스의 하위 요인 중 ‘사회적 거리두기로 인한 어려움(r=.27, *p*=.001)’과 약한 양의 상관관계가 있었다. 그리고 COVID-19에 대한 두려움은 COVID-19 스트레스 하위 요인 ‘사회적 거리두기로 인한 어려움(r=.46, *p*<.001)’, ‘감염에 대한 두려움(r=.31, *p*<.001)’, ‘타인에 대한 분노(r=.24, *p*=.002)’와 양의 상관관계가 있었다(Table 4).

### 우울에 미치는 영향 요인

임부의 우울에 영향을 미치는 요인을 확인하기 위해 단변량 분석에서 통계적으로 유의한 차이를 보인 8개의 변수를 투입하였다. 임부의 COVID-19에 대한 두려움, COVID-19 스트레스의 하위 영역인 감염에 대한 두려움, 사회적 거리두기로 인한 어려움 및 타인에 대한 분노 등은 연속 변수로 입력하였고, 대상자의 특성 중 임신 분기(2삼분기, 0; 3삼분기 1), 계획 임신 여부(계획 임신, 0; 비계획 임신, 1), COVID-19 확진자와 접촉 유무(없는 경우, 0; 있는 경우, 1), 삶의 제약 유무(없는 경우, 0; 있는 경우, 1) 등은 더미 변수로 처리하여 동시적 다중회귀분석을 시행하였다.

회귀분석에 앞서 다중 공선성 여부를 확인한 결과 공차 한계가 0.57–0.90으로 0.1 이상이었고 분산팽창지수(variation inflation factor)는 1.11–1.75로 10을 넘지 않아 다중 공선성 문제는 없었으며 변수 간의 상관계수가 0.1–0.6으로 0.8을 넘지 않아 예측 변수 간의 독립성이 확보되었다. Durbin-Watson값은 2.21로 2에 가까워 자기 상관이 없었고, 잔차의 분포가 균등하게 흩어져 있는 산점도와 정규분포 모양의 표준화 잔차 정규 p-p 도표로 통해 회귀식의 가정이 모두 만족되었음을 확인하였다.

임부의 우울에 가장 크게 영향을 미친 요인은 COVID-19에 대한 두려움(β=.26, *p*=.002)이었고, 다음은 임부가 COVID-19 확진자와 접촉한 경우(β=.21, *p*=.004), 임신 3삼분기(β=.18, *p*=012), COVID-19 스트레스의 하부 요인인 ‘사회적 거리두기로 인한 어려움’으로 스트레스가 높은 경우(β=.16, *p*=047), 비계획 임신인 경우(β=.15, *p*=042) 순이었다. 본 연구의 회귀 모형은 통계적으로 유의하였고(F=7.73, *p*<.001), 이 모형은 35.0%의 설명력을 보였다(Table 5).

## Discussion

본 연구는 COVID-19로 인해 전 세계인들이 어려움을 겪고 있는 상황에서 새로운 생명을 품고 지켜야 하는 임부를 대상으로 이들의 심리적 문제를 파악하고, 중재방안을 마련하기 위해 시도하였다.

본 연구에서 임부의 우울에 영향을 미치는 요인은 COVID-19에 대한 두려움, COVID-19 확진자와의 접촉 경험, 임신 3삼분기, 사회적 거리두기로 인한 어려움, 비계획 임신 순이었다.

본 연구에서 임부의 우울은 14.86점이었고 대상자 중 약 50%가 우울을 경험하였다. 본 연구에서 우울을 측정하기 위해 사용한 K-CESD-R은 DSM 4판의 우울 증상을 반영하여 개정한 CES-D-R을 2016년에 한국인 대상으로 표준화한 것으로[27], 본 도구를 사용하여 팬데믹 전후 국내 임부의 우울 유병률에 대해 같은 도구를 사용한 연구가 없어 직접 비교하기는 어렵다. 그러나 원 도구(CES-D-R)에서 제시한 점수 기준에 따라[31] 총점을 60점으로 변환하고 16점을 절단점으로 하였을 때 본 연구에서 우울 임부는 43.8%였고, 이를 팬데믹 이전 원 도구의 기준을 사용한 국내 임부 유병률 35.3% [1]와 비교해 볼 때 팬데믹 이전보다 이후에 임부의 우울이 증가했다고 판단할 수 있다. 같은 도구를 사용하여 캐나다 임부를 대상으로 한 연구에서도 COVID-19 팬데믹 동안 임부의 57.3%가 우울을 경험한 것으로 나타났다[32]. COVID-19 팬데믹 동안 23편의 연구를 메타 분석한 연구에서도 임부의 불안, 우울, 심리적 스트레스 등의 정신질환 유병률은 각각 37%, 31%, 70%로 높았으며, 임부의 불안과 우울의 상대위험도는 COVID-19 팬데믹 전과 비교하여 각각 1.65와 1.08로 나타났다[13].

COVID-19에 대한 두려움은 신종 감염성 질환에 대한 불확실성과 바이러스로 인해 나타나는 불안한 감정으로[33], 본 연구에서 임부의 COVID-19에 대한 두려움은 평균 21.5점으로 중간 이상 수준이었고, 튀르키예 임부의 COVID-19에 대한 두려움(21.6점)과 비슷하였다[18]. 또한 임부의 COVID-19에 대한 두려움은 일반 성인 18.2점[24]과 일반 여성 17.8점[34]보다 더 높은 것으로 나타났다. 임부의 COVID-19 감염은 폐기능 악화로 인한 중환자실 입실 위험과 조산 및 제왕절개 분만을 초래하여[14] 태아의 건강에 부정적인 영향을 미칠 수 있다는 데 대한 염려와 두려움의 반응으로[35], 일반인보다 COVID-19에 대한 두려움이 더 높은 것으로 생각된다.

COVID-19에 대한 두려움과 우울은 팬데믹 동안 임부가 경험하는 일반적인 심리적 문제로, Fan 등[10]과 Durmuş 등[18]의 연구에서도 두려움과 우울은 서로의 영향 요인으로 나타났다. COVID-19 확진자와 사망자의 급속한 증가는 COVID-19에 대한 두려움을 유발하고, 바이러스에 대한 불확실성 및 불안전한 일상에 대한 불안감으로 팬데믹 동안 임부는 우울을 경험하는 것으로 확인되었다[18]. 그리고 본 연구 조사 시에는 오미크론 변이 바이러스의 확산을 통해 확진자가 급증함에 따라 의료체계 유지를 위해 재택 치료 체계로 전환하였다[11]. 재택 치료 전환 시 응급 환자의 이송체계나 검사체계가 준비되지 않아 일차 의료기관의 혼란을 야기하였고, 보건소에 의존한 재택 치료는 원활한 환자 관리가 부족한 실정이었다. 이에 임부들은 진통이나 출산 등 갑작스러운 응급상황 발생 시의 병원 진료 체계에 대한 정보 부족과 불안감으로 더욱 두려움을 경험한 것으로 보인다.

임부의 COVID-19에 대한 두려움과 불안은 신종 감염병 바이러스에 대한 정확하고 만족할 만한 정보 제공으로 완화할 수 있는데[36], 본 연구에서 임부는 COVID-19와 관련된 정보를 대부분 TV, 라디오, 신문과 같은 미디어와 인터넷 블로그를 통해 얻고 있었고, 스페인의 임부도 인터넷(72.8%)이나 소셜 미디어(31.4%)와 같이 비교적 접근이 쉬운 비공식 사이트를 통해 얻고 있었다[36]. 비공식 사이트를 통한 정보 수집은 잘못된 정보의 확산과 함께 임부의 두려움과 불안을 더욱 증가시킬 수 있어 질병관리청과 같은 공식 사이트 이용 안내와 산전 진료 시 전문 의료진의 적절한 정보 제공이 필수적이다.

임부의 COVID-19 확진자와의 접촉 경험은 우울에 영향을 미치는 요인으로, 확진자 접촉이 태아와 가족 등 주변 사람들에게 미치는 영향에 대한 지속된 염려가 임부의 불안과 우울을 유발하는 것으로 나타났다[12]. 본 연구에서 임부는 본인의 감염에 대한 걱정보다 태아에 대해 더 염려하는 것으로 확인하였고, 임부는 태아의 건강을 우선 순위로 생각하며 COVID-19 확진자와의 접촉으로 본인이 감염되거나 격리하는 동안 태아에게 발생할 수 있는 위험한 상황에 대한 염려로 불안과 우울이 나타나는 것으로 보인다. 의료진은 산전 진료 시 임부의 염려 사항을 파악하여 감염 발생 시의 행동지침과 진료 체계에 대해 안내해 주어야 하고, 정부는 자가격리 중인 임부를 대상으로 감염 증상에 대한 평가와 함께 제한된 사회적 지지체계 내에서 임부를 위한 심리적 지원 대책이 필요하다.

본 연구에서 임신 3삼분기도 우울의 영향 요인인 것으로 확인하였다. 임신 3삼분기는 임부가 태아와 일체감을 가지며 출산 후 모성 역할과 분만에 대한 두려움을 경험하는 시기로, 임신 분기의 진행 정도에 따라 임부가 경험하는 피로가 증가하면서 이에 따른 신체적, 정신적 스트레스로 임부의 우울이 높은 것으로 알려져 있다[37]. 이러한 결과는 팬데믹 이전이나 이후 모두 임신 3분기는 우울에 취약한 기간이므로 임부를 위한 정신건강관리 프로그램을 계획할 때 특히 이 시기 임부에 대한 관심이 필요하다는 것을 시사한다.

그리고 본 연구 결과 COVID-19 스트레스의 하위 요인 중 ‘사회적 거리두기로 인한 어려움’이 임부의 우울과 약한 양의 상관관계가 있는 것으로 나타났고, 감염 확산을 통제하기 위한 사회적 거리두기와 같은 사회적 조치가 임부의 우울에 영향을 미치는 것으로 확인되었다. 한국 문화 특성인 관계 중심의 신체활동과 사회적 지지는 임부의 우울을 완화하는 요인이다[24]. 그러나 반복적인 COVID-19 유행 상황에 따른 사회적 거리두기 장기화는 임부의 대면 상호작용 및 문화 활동 감소를 초래하였고, 사회적 거리두기와 같은 비일상적인 경험은 주산기 활동을 통한 임부의 삶의 의미 감소와 무기력감, 스트레스를 가중시켜[19] 임부의 우울이 증가한 것으로 보인다. 의료진과 보건 당국은 사회적 거리두기에 영향을 받지 않고 임부와 배우자, 의료진 및 다른 임부들이 함께 참여하고 활동할 수 있는 산전 온라인 프로그램을 활성화하고 적극적인 홍보와 안내를 할 필요가 있다.

임부 우울의 마지막 영향 요인은 비계획 임신이었다. 여성은 임신 전 건강 관리와 함께 양육을 위한 경제적 능력, 모성 역할에 대한 마음가짐 등의 준비가 필요하지만, 비계획 임신은 의도하지 않은 상황으로 인해 임부의 스트레스를 유발하며 산전 관리를 위한 약물 및 음주 복용을 중단하지 못한 것 등 태아에게 미치는 영향에 대한 죄책감이 우울을 유발한다[24]. 튀르키예 임부를 대상으로 팬데믹 이전에 벡 우울척도(Beck Depression Inventory)로 측정한 비계획 임신부의 우울은 10점이었고[38], 팬데믹 이후 비계획 임신부의 우울은 13점으로 나타나[39] 팬데믹 후의 비계획 임신이 팬데믹 이전보다 우울 점수가 증가한 것으로 볼 수 있다. 비계획 임신은 임부에게 갑작스러운 모성 역할과 태아 애착에 대한 감정 변화, 개인의 신체 변화, 사회·경제적 상태 변화를 초래하고 COVID-19 팬데믹은 더욱 예측하기 어려운 일상 변화와 감염에 대한 두려움, 스트레스를 유발하여 비계획 임신부의 우울이 더욱 악화하는 것으로 보인다.

COVID-19 스트레스 정도는 하위 요인에 따라 다르게 나타났는데, 하위 요인 중 ‘타인에 대한 분노’가 가장 큰 스트레스 하부 요인(4.16점)인 것으로 확인되었다. 동일한 도구로 측정한 간호대학생의 COVID-19 스트레스도 타인에 대한 분노(4.08점)가 가장 큰 스트레스 요인이었으나[40], 임부의 점수보다는 낮았다. 타인에 대한 분노는 한 개인이 COVID-19 감염 확산을 줄이기 위해 손 위생, 마스크 착용, 모임 자제 등 정부 지침에 따른 예방 수칙을 철저히 지키는 반면, 감염의 위험을 증가시키는 타인에 대해 나타나는 분노의 반응으로[41], 국내 COVID-19 발생 초기 확진자 동선 공개 및 언론보도에 따른 감염병 확산 원인에 대한 분노와 비난이 반영되어 나타난 결과로 볼 수 있다[19]. 그리고 팬데믹 발생은 임부 스스로 통제할 수 없는 상황으로, 임부는 본인과 태아에 미치는 영향에 대한 걱정과 함께 특정 집단 및 개인에 대한 분노와 스트레스가 다른 사람들보다 높은 것으로 생각된다.

그리고 본 연구 대상자가 COVID-19로 인해 겪었던 가장 힘든 경험은 ‘산전 진료 시 보호자의 출입 제한’이었다. 배우자는 산전 진료 및 교육 참여를 통해 임부가 경험하는 신체적, 정서적 불편감에 대한 이해와 공감이 높아지고, 부부간의 긍정적인 소통을 통해 건강한 모성 정체성을 가질 수 있도록 지지체계를 형성한다[24]. 임신 과정에서 배우자의 지지는 중요한 스트레스 완화 요인이며 임부에게 심리적 안정감을 제공하여 긍정적인 영향을 미치는데[42], 사회적 거리두기로 인하여 병원 내 보호자 출입이 제한되면서 산전 진료 과정에서 겪는 심리적 고립감과 두려움을 임부 혼자서 경험해야 하는 것이 힘들었던 것으로 보인다. 감염의 확산을 예방하기 위해 최소 인원의 접촉과 거리두기가 중요한 방역 대책이지만[9,10], 임부에게 정서적 안정감을 제공하고 임신 과정의 경험을 긍정적으로 배우자와 공유하기 위해 이를 고려한 진료‧방역지침이 마련되어야 한다.

본 연구는 사회적 감염병에 대한 두려움, 확진자와의 접촉, 그리고 사회적 거리두기 조치로 인한 스트레스가 COVID-19 유행 상황 시 임부의 우울에 영향을 미치는 요인임을 확인하여 추후 신종 감염병 발생 시 임부를 위한 간호중재 프로그램 개발을 위한 기초적인 자료를 제공한 것에는 의의가 있으나, 몇 가지 제한점이 있다. COVID-19 팬데믹이라는 사회적 상황이 임부의 우울에 어떠한 영향을 미치는지 확인하기 위한 연구이므로, 임신 중 우울의 주 영향 요인인 우울 과거력[43]이 있는 임부는 제외하여 우울의 영향 요인에 대한 설명력이 35%로 다소 낮았다. 또한 COVID-19 팬데믹은 감염병 확산이 전 세계에 영향을 미치는 일시적인 현상으로 각 나라 및 지역마다 감염병 유행 상황이 달라 COVID-19 관련 특성과 임부의 우울은 측정 시기와 문화에 따라 다를 수 있다는 제한점이 있다

결론적으로 신종 감염병의 대유행 시 임부의 정신건강은 산전 관리 중에 평가되어야 하고, 전염병에 대한 정확한 정보와 임부의 안전을 위한 행동지침을 제대로 교육하여 임부의 두려움을 완화할 필요가 있다. 그리고 임부의 사회적 활동을 위한 비대면 교육 프로그램을 활성화하여 가벼운 실내 활동, 타인과의 지속적인 소통을 유지하여 ‘사회적 거리두기’로 인한 임부의 스트레스를 완화할 필요가 있다.

## Figures and Tables

**Table 1.** Differences in prenatal depression according to participants’ characteristics (N=153)

Variable	Categories	Mean±SD or n (%)	Prenatal depression	
			Mean±SD	t/F (*p*)
Age (year)		32.80±3.55		
	<30	24 (15.7)	15.79± 9.80	0.97 (.381)
	30–34	80 (52.3)	15.70±12.03	
	≥35	49 (32.0)	13.04±10.06	
Education level	≤High school	12 (7.8)	15.75± 5.03	0.55 (.586)
	≥College	141 (92.2)	14.79±11.48	
Employed	Yes	112 (73.2)	15.07±11.29	0.38 (.702)
	No	41 (26.8)	14.29±10.71	
Monthly household income (Korean won^†^)	<3 million	28 (18.3)	13.82±11.14	0.23 (.794)
	3-4.9 million	65 (42.5)	14.71±10.93	
	≥5 million	60 (39.2)	15.52±11.41	
Trimesters (week)		26.68±6.33		
	2nd (14–28)	78 (51.0)	12.55±10.12	–2.68 (.008)
	3rd (>28)	75 (49.0)	17.27±11.63	
Gravidity	Primigravida	108 (70.6)	14.68±10.40	0.32 (.748)
	Multigravida	45 (29.4)	15.31±12.74	
Intended pregnancy	Yes	104 (68.0)	13.00±10.33	–3.11 (.002)
	No	49 (32.0)	18.82±11.74	
Prenatal care	Regular	123 (80.4)	14.71±11.12	–0.15 (.885)
	Irregular	30 (19.6)	15.03±11.32	
Contact with a confirmed COVID-19 case	Yes	24 (15.7)	21.33±12.61	–3.20 (.002)
	No	129 (84.3)	13.66±10.42	
Life constraints	Yes	141 (92.2)	14.20±11.11	3.49 (.003)
	No	12 (7.8)	22.67± 7.75	
Preferred delivery method	Vaginal delivery	89 (58.2)		
	Cesarean section	64 (41.8)		
Sources of information related to COVID-19	Official website (KDCA)	13 (8.5)		
	Media (TV, radio)	61 (39.9)		
	Internet	54 (35.3)		
	Social network service	22 (14.3)		
	Acquaintance	3 (2.0)		
Trust in information	Yes	109 (71.2)		
	No	44 (28.8)		

COVID-19: Coronavirus disease 2019; KDCA, Korean Disease Control and Prevention Agency.

† One million Korean won is roughly 800 US dollars.

**Table 2.** Scores for uncomfortable experiences and concerns due to COVID-19 (N=153)

Variable	Categories	Mean±SD
Uncomfortable experiences due to COVID-19	Restriction on spouse’s access during prenatal care	4.10±1.64
	Restriction on going out	3.85±1.74
	Coronavirus vaccination restrictions	3.82±1.59
	Reduced events and education on pregnancy and childbirth information	3.46±1.59
	Wearing mask	3.40±1.69
	Getting tested for COVID-19 before childbirth	2.39±1.49
Concerns related to COVID-19	I’m worried about effect of COVID-19 on the unborn baby.	4.08±1.03
	I’m worried about getting infected with COVID-19.	3.67±1.29
	I’m worried that complications of pregnancy will occur because I cannot receive prenatal care due to COVID-19.	2.58±1.23
	I am concerned about the spread of the coronavirus to other people (family, acquaintances, etc.).	2.37±1.28
	I'm concerned about being infected with COVID-19 from the hospital environment during treatment or delivery.	2.29±1.21

COVID-19: Coronavirus disease 2019.

**Table 3.** Levels of prenatal depression, fear of COVID-19, and COVID-19 stress (N=153)

Variable	Categories	n (%)	Possible range	Mean±SD (range)
Prenatal depression	<13	76 (49.7)	0–80	14.86±11.10 (0–45)
	≥13	77 (50.3)		
Fear of COVID-19			7–35	21.55±4.90 (9–33)
COVID-19 stress	Fear of infection		1–5	4.00±0.51 (2.67–5.00)
	Difficulties of social distancing		1–5	3.41±0.69 (1.00–4.83)
	Anger toward others		1–5	4.16±0.69 (1.83–5.00)

COVID-19: Coronavirus disease 2019.

**Table 4.** Relationships among prenatal depression, fear of COVID-19, and COVID-19 stress (N=153)

Variable	Categories	r (*p*)				
		Prenatal depression	Fear of COVID-19	COVID-19 stress		
				Fear of infection	Difficulties of social distancing	Anger toward others
Fear of COVID-19		.38 (<.001)	1			
COVID-19 stress	Fear of infection	.02 (.848)	.31 (<.001)	1		
	Difficulties of social distancing	.27 (.001)	.46 (<.001)	.34 (<.001)	1	
	Anger toward others	.07 (.405)	.24 (.002)	.60 (<.001)	.34 (<.001)	1

COVID-19: Coronavirus disease 2019.

**Table 5.** Factors influencing prenatal depression among the participants (N=153)

Variables	B	SE	β	t	*p*
(constant)	3.05	6.96		0.44	.662
Fear of COVID-19	0.60	0.19	.26	3.21	.002
Fear of infection	–0.26	0.22	–.11	–1.21	.241
Difficulties of social distancing	0.43	0.21	.16	2.00	.047
Anger toward others	0.011	0.23	.01	0.05	.962
Trimester^†^	4.03	1.57	.18	2.56	.012
Intended pregnancy^†^	3.53	1.72	.15	2.06	.042
Contact history with a confirmed patient^†^	6.37	2.19	.21	2.91	.004
Life constraints^†^	4.98	3.21	.12	1.55	.123
R^2^=.35, adjusted R^2^=.31 F=7.73 (*p*<.001)					

COVID-19: Coronavirus disease 2019.

† The reference variables were trimester (second trimester), intended pregnancy (yes), contact history (no), and life constraints (no).
